# Lung Cancers Associated with Cystic Airspaces

**DOI:** 10.3390/cancers17020307

**Published:** 2025-01-18

**Authors:** Clara Valsecchi, Francesco Petrella, Stefania Freguia, Milo Frattini, Gianluca Argentieri, Carla Puligheddu, Giorgio Treglia, Stefania Rizzo

**Affiliations:** 1 Clinic of Radiology EOC, Istituto Imaging della Svizzera Italiana (IIMSI), Via Tesserete 46, 6900 Lugano, CH, Switzerland; gianluca.argentieri@eoc.ch (G.A.); carla.puligheddu@eoc.ch (C.P.); stefania.rizzo@eoc.ch (S.R.); 2 Department of Thoracic Surgery, Fondazione IRCCS San Gerardo dei Tintori, 20900 Monza, IT, Italy; francesco.petrella@irccs-sangerardo.it; 3 Istituto Cantonale di Patologia EOC, Via in Selva 24, 6600 Locarno, CH, Switzerland; stefania.freguia@eoc.ch (S.F.); milo.frattini@eoc.ch (M.F.); 4 Faculty of Biomedical Sciences, Università della Svizzera italiana (USI), Via G.Buffi 13, 6900 Lugano, CH, Switzerland; giorgio.treglia@eoc.ch; 5 Clinic of Nuclear Medicine, Istituto Imaging della Svizzera Italiana (IIMSI), Via Tesserete 46, 6900 Lugano, CH, Switzerland; 6 Faculty of Biology and Medicine, University of Lausanne, 1011 Lausanne, CH, Switzerland

**Keywords:** lung cancer, cystic airspaces, diagnosis, treatment

## Abstract

Lung cancer, the second most common malignancy in both men and women, poses a significant health burden. Early diagnosis remains pivotal in reducing lung cancer mortality. Given the escalating number of computed tomography (CT) examinations in both outpatient and inpatient settings, radiologists play a crucial role in identifying early-stage pulmonary cancers, particularly non-nodular cancers. Screening programs have been instituted to achieve this goal, and they have raised attention within the scientific community to lung cancers associated with cystic airspaces. These cancers, although they have been known for at least a decade, remain understudied. Limited investigations with small sample sizes have estimated their prevalence and explored their radiological and pathological features. Lung cancers associated with cystic airspaces exhibit varying complexities within their cystic components and demonstrate suspicious changes over time. Adenocarcinoma is the predominant histological type, often with a peripheral location. Differential diagnosis on CT scans includes inflammatory processes or emphysema-related changes. Unfortunately, prospective studies specifically analyzing the prevalence of cystic airspace-associated lung cancers are lacking. However, it is estimated that they constitute approximately one-fourth of delayed radiological diagnoses. Increased awareness among radiologists could lead to more timely identification and potentially reduce lung cancer mortality in a cost-effective manner.

## Simple Summary

Lung cancer, the second most common cancer in both men and women, is a major health issue, and early detection is key to reducing deaths from the disease. Radiologists, who read CT scans, play an important role in spotting lung cancers early, even those that are not clear nodules. One particular type of lung cancer, associated with cystic airspaces, has received growing attention in research, though it remains under-studied. These cancers, mainly adenocarcinomas, often appear in the outer parts of the lungs and can be confused with other pathologies like infections or emphysema. While it is estimated that lung cancers associated with cystic airspaces account for about a quarter of lung cancer diagnoses that are missed or delayed, more research is needed to better understand them. Raising awareness about these types of cancer could help catch them earlier and potentially save lives, all while being a cost-effective approach.

## 1. Introduction

Lung cancer is the second most common malignancy worldwide, affecting both men and women, and remains the leading cause of cancer-related mortality globally [1]. It is most often detected through computed tomography (CT), typically presenting as pulmonary nodules or masses. Unfortunately, most lung cancer cases are diagnosed at advanced stages, which correlate with a poor prognosis. The most effective strategies to reduce lung cancer mortality include public education on the health risks associated with smoking, particularly targeting younger populations, along with smoking cessation and screening programs with low-dose CT (LDCT). Behavioral interventions, though challenging to implement, have the greatest long-term impact.

On the one hand, in the last twenty years, large-scale screening studies with low-dose CT in individuals with defined risk factors, mostly including current or former smokers and aged from 50 to 70 years, have demonstrated a significant reduction in lung cancer mortality [2,3]. On the other hand, LDCT screening itself carries potential harms, such as overdiagnosis and overtreatment. Moreover, its practical implementation is hindered by economic, organizational, and psychological barriers. Some experts have suggested that a more cost-effective and safer approach might be personalized screening, in which recruitment and follow-up intervals are tailored to the individual’s risk profile including blood testing [4].

Furthermore, the demand for radiological studies, including chest CT scans, has increased in both emergency settings and specialized oncological practices [5], due to defensive medicine and the widespread availability of CT imaging in many developed countries. At the same time, radiology has become more specialized, with a shift towards organ-specific subspecialty training, giving rise to the advantage of delivering better reports to the referring clinicians and thus improving patient care quality [6,7]; however, this sub-specialization can make it difficult, if not impossible, for radiologists to stay updated across all sub-disciplines.

In this landscape, the interpretation of lung cancer associated with cystic airspaces may be problematic for non-expert radiologists, as well as for referring physicians and oncologists, who may not be aware of the risks that these cystic airspaces are associated with.

The concept of lung cancers associated with cystic airspaces has been described in the literature since 1940, when a surgical pathology study suggested that congenital lung cysts might represent a risk factor for the development of epithelial metaplasia [8]. In the decades that followed, several studies sought to clarify the relationship between cystic airspaces and lung cancer, but consensus regarding a unique precise definition and pathogenesis has remained unclear [9,10,11]. In the last two decades, screening studies with LDCT have provided a larger amount of data, giving more insight into the morphological characteristics and temporal evolution of cystic lung cancers and contributing to a more robust understanding [12]. By promptly identifying suspicious lung cancers—ranging from classical nodules to less typical cancers associated with cystic airspaces—radiologists can contribute to early detection and management, acting as part of an opportunistic screening strategy.

This review aims to summarize the current understanding and ongoing studies regarding lung cancer associated with cystic airspaces.

## 2. Lung Cystic Airspaces

Benign lung cysts are estimated to occur in 8% of individuals over the age of 40 [13].

Causes of cystic lung disease can be different; there are congenital forms, smoking-related forms (Langerhans cell histiocytosis, emphysema), lymphangioleiomyomatosis, the cystic form of lymphocytic pneumonia, forms secondary to amyloidosis, and forms secondary to infection or trauma. In addition, some cancers may have a partially cystic appearance. Little is known about the early indicators suggesting that a cystic airspace is associated with lung cancer, and the exact carcinogenic mechanisms remain unclear. Several theories have been proposed regarding the development of cystic airspaces in lung cancer. Most cystic airspaces are thought to result from a “check-valve” mechanism, which may explain their progressive enlargement over time. This mechanism could be due to the extrinsic compression of small airways by fibrous tissue deposition produced by lung cancer or by direct cancer extension into the airway. Another proposed mechanism involves cystification, a process where cancer degeneration caused by vascular insufficiency leads to the resorption of degenerated tissue. Distinguishing between these different types of cystic airspaces on CT can be challenging, which further complicates the diagnosis of lung cancer in cases involving cystic airspaces.

Additionally, in a small subset of patients with congenital cystic malformations, these represent a recognized risk factor for malignancy, even in young subjects, particularly mucinous adenocarcinoma. A study demonstrated that proliferations of mucinous cells within type I congenital pulmonary airway malformations exhibit a genetic profile similar to that of mucinous adenocarcinomas of the lung [14].

Lung cancers with cystic airspaces in the vast majority of cases turn out to be adenocarcinomas based on histology. This knowledge derives from the studies published so far on cohorts that included a number of patients ranging between 300 and 350. In a minority of cases, this radiological presentation corresponds to another histology (squamous, adenosquamous, undifferentiated) [15]. Another interesting fact is the hypothesis that poorly differentiated adenocarcinoma often shows a specific radiological correlation, such as cystic airspaces with a mural nodule [16]. However, this frequent association remains to be verified with larger samples. The histological analyses carried out so far have also provided useful information regarding several possible pathogenetic mechanisms.

At the molecular level, available data are still scarce, and among the few studies that have considered this aspect, they report epithelial growth factor receptor (EGFR) and kirsten rat sarcoma virus (KRAS) mutations and ALK rearrangements as the most frequent alterations [17] thus hypothesizing that the molecular profile of cystic airspace-associated lung cancers does not differ substantially from the molecular picture of adenocarcinoma, although the number of reported cases is too small to draw any firm conclusion on this issue. On the other hand, it should be reported that, again in a very limited cohort, a substantial proportion of lung cancers associated with cystic airspace seems to be related to ALK rearrangements rather than EGFR-mutant cases [18].

## 3. CT Features

### 3.1. CT Protocol

The main tool for the diagnosis of lung cystic airspaces and their evolution towards lung cancer is chest CT, in which the evaluation of the multiplanar reconstructed images allows us to evaluate at best the possible changes that these lung cystic airspaces may undergo over time. It is also important to consider that lung cystic airspaces are prone to a wide variability in their measurement and evaluation, especially as they may show dimensional and morphological changes in either their solid or cystic parts or both. Until today, there have been no dedicated studies which compare a standard-dose CT and a low-dose CT in diagnostic performance of lung cancer associated with cystic airspaces. Instead, many studies have found that there is no significant clinical difference in the diagnostic accuracy of lung nodules (solid and ground-glass) detection and characterization between a standard-dose CT and a low-dose CT [19,20]. For this reason, lung cancer screening programs require low-dose CT scans, and there is promising research regarding ultra-low-dose chest CT for this kind of lung pathologies [21]. Therefore, it could be assumed that for lung cancer associated with cystic airspaces, an LDCT can have the same or very similar accuracy to that of a normal-dose chest CT, as cancerous tissue has higher attenuation than normal lung parenchyma. In this landscape photon counting CT (PC-CT) has great potential thanks to its higher resolution at lower doses, as demonstrated in multiple studies [22]. PC- CT allows for a more detailed lung parenchyma analysis at equal or lower radiations doses than routine chest CT thus offering an ideal diagnostic tool both for patients and radiologists, especially when a follow-up CT is necessary. Unfortunately, specific studies that clearly analyze diagnostic performance of PC-CT in lung cancer associated with cystic airspaces are lacking. 

The administration of intravenous contrast is not specifically recommended for evaluating lung cystic airspaces, similarly to lung ground glass nodules, due to the satisfactory high contrast to noise ratio between them and the background lung parenchyma. The main and most widely accepted indications for administration of contrast in chest CT are lung cancer staging and follow-up, mediastinal structure evaluation, pleural diseases such as empyema, and suspected mesothelioma [23].

### 3.2. Definition

The definition of “cystic airspace” is relatively recent, compared to other well-established thoracic radiology terms, such as solid, ground-glass, or part-solid nodules. It can be an ambiguous term because there is a wide variety of lung lesions that can be classified as cystic airspaces, as well as from their subjective interpretation on imaging.

Common CT findings in this category include congenital cysts, emphysema bullae, fibrotic cysts, and the dilation of distal airways, often resulting from cancerous obstructive phenomena. Recent updates to thoracic radiology terminology briefly addressed this issue, officially redefining the term “cystic” as a descriptor for lesions characterized by central air-equivalent attenuation, surrounded by a wall of variable thickness and regularity [24]. Importantly, this definition does not imply any etiology, given that the underlying cause of cystic airspaces is often indeterminate on CT.

Lung cancer associated with cystic airspaces is a type of lung cancer characterized by the presence of cystic cavities in or around the solid component observed during imaging.

**Figure 1:**
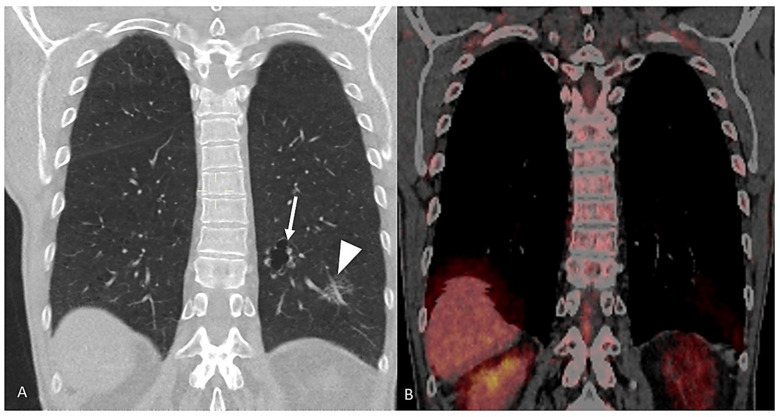
Coronal chest CT (**A**) shows two adenocarcinomas in the left lower lobe, one with the appearance of an atypical cyst (white arrow) and the other one with the appearance of a partially solid nodule (white arrowhead). PET-CT (**B**) of the same patient does not show any significant metabolic activity of the two lung nodules.

**Figure 2:**
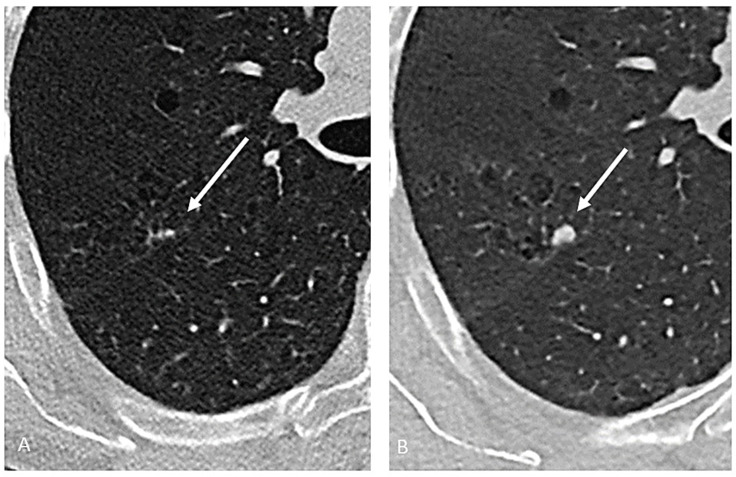
Axial chest CT showing a small area of increased density in the right superior lobe, adjacent to cystic emphysematous area (**A**), that increased in size and density after one year (**B**) and was proven to be an adenocarcinoma.

The radiological appearance of lung cancer associated with cystic airspaces is variable, but it usually includes specific features, such as the presence of a cystic airspace associated with a thickening of the wall, which is often irregular but sometimes nodular (Figure 1), or the presence of areas of increased density of the lung parenchyma, both consolidative and ground-glass, adjacent to the airspaces (Figure 2). The advent of lung cancer associated to cystic airspaces usually present as a new development of diffuse nodularity, eccentric nodules or ground glass change associated with the cystic airspace [25]. The lung cancers arising in association with cystic airspaces are most commonly adenocarcinomas based on histology.

A cavity is a different entity compared to cystic airspaces, being a gas-filled space and seen as a lucency or low-attenuation area within pulmonary consolidation or within a mass or a nodule [24]. In contrast to cavities or lung masses with a cavity which can sometimes contain a certain fluid level, the cystic part of lung cancer associated with cystic airspaces does not contain fluid. In cases where the diffuse wall thickening of a cystic airspace progresses (type III, as explained in the classification paragraph), however, it may mimic a lung cancer associated with cystic airspaces.

### 3.3. Classification Systems

In the last decade, different morphological classifications of lung cystic airspaces have been found to combine in a variable way. The following characteristics have been proposed: For example, in 2015, Mascalchi et al. proposed a classification into four types, namely type I cystic lesion with an exophytic nodule, type II cystic lesion with an endophytic nodule, type III cystic lesion with a parietal thickening, and type IV multiloculate cystic lesions [26]. Subsequently, Fintelaman developed a more articulated classification divided into three subcategories, namely cystic spaces (thin-walled or thickened, with exo or endophytic nodules), cystic lesions (uni- or multiloculated), and nodular portions (solid-density, ground-glass, or mixed) [27]. More recently, Shen et al. developed a simplified classification into the following four categories: I (thin-walled cystic lesion), II thickened-walled cystic lesion, III cystic lesions with nodule (exo or endophytic), and IV multiloculated cystic lesion with solid/subsolid portions [16]. However, no study has shown an association between the different morphological types and different prognoses. Several authors also believe that the different morphologies described can represent different stages of the same pathogenetic process. For example, in some studies, the growth of thickness or the appearance of a nodule, the change in the cystic appearance, from uni-to multiloculate have been recognized as suspicious signs of evolution towards cancer [16,27].

The Lung CT Screening Reporting and Data System (Lung-RADS) are the criteria for the radiological classification of lung lesions into distinct categories from 0 to 4, depending on the growing suspicion of their cancerous nature. Created in 2014 by the American College of Radiology (ACR), the system helps to make the classification and management of pulmonary nodules in screening unambiguous [28]. The first update was made in 2019, reviewing the size thresholds of subsolid nodules and adding some details on the various types of nodule morphology (version 1.1). The second update took place more recently, in 2022, and among the changes made in this current version, specific cystic airspaces were introduced in a standardized way, with the nomenclature of atypical lung cysts [29]. An atypical lung cyst according to the lung-RADS is a cyst showing a wall with a thickness equal to or greater than 2 mm, which has uniform or asymmetrical morphology, uni-or multiloculate, with possible parietal nodules. They were introduced into different categories, 3, 4a, and b according to their different morphological characteristics and temporal progression. Category 3 (probably benign, follow-up at 6 months) includes atypical lung cysts, i.e., cysts with thick walls that show a dimension growth of their cystic part. Category 4A (suspicious, follow-up at 3 months) includes cysts with thick walls or multiloculated at baseline or cysts that become multiloculated, regardless of their thickness parietal). Category 4B (very suspicious, diagnostic work up) includes cysts with parietal thickening at baseline and growth over time, with the increase in size of multiloculated cysts, which acquire new internal septa plus loculates or with the appearance of solid or drooping portions adjacent to them [29]. Notably, the following entities are not included in the lung-RADS classification: uniculate thin-walled cysts with a wall thickness < 2 mm, which should be considered benign; multiple cysts that may indicate diffuse cystic lung disease; cysts containing fluid levels as they may represent infectious processes.

Table 1 shows CT findings and management of atypical pulmonary cystic airspaces according to the last Lung RADSv2022 [29].

### 3.4. Risk of Malignancy

Lung cancer associated with cystic airspaces is deemed less common than more typical nodules or masses, but data on its incidence and prevalence are limited. Most estimates are based on retrospective studies of screened populations, which have evaluated the percentage of cases with cystic airspaces and the morphological characteristics of missed or late diagnoses. For instance, a 2012 study from the early lung cancer action program (ELCAP) estimated the prevalence of lung cancer associated with cystic airspaces at approximately 3.7% [12]. A few years later, the Dutch–Belgian lung cancer screening trial (Nederlands–Leuvens Longkanker Screenings Onderzoek –NELSON) study reported that lung cancers associated with cystic airspaces accounted for 22% of late diagnoses, comparable to endobronchial cancers [30]. A study of 441 patients diagnosed with primary lung cancer found that 9.3% were associated with cystic airspaces at initial CT imaging [31]. A systematic review including only retrospective studies, reports some epidemiological information such as average age at diagnosis of about 62, slight prevalence in men compared to women and in smokers compared to non-smokers [15]. Currently a prospective study analyzing the incidence and prevalence of cystic airspaces is lacking.

In case cystic airspaces are diagnosed as malignant, their staging should follow the standard tumour nodes metastases (TNM) staging for lung cancers, currently on the way to the application of the 9th edition (starting January 2025) [32].

### 3.5. Differential Diagnosis

Lung cancers associated with cystic airspaces are mainly differential diagnoses with infectious–inflammatory processes or with parenchymal changes due to emphysema or interstitial disease/fibrosis. Less frequent differential diagnoses include cystic metastases, cysts associated with systemic disorders such as amyloidosis. The unequivocal distinction between infectious inflammatory process and lung cancers with cystic airspaces is often not possible with a single CT scan, particularly if the lung parenchyma is affected by emphysema or fibrosis, conditions that predispose to the development of inflammatory and infectious processes, other than cancers. In any case, some clinical-anamnestic and radiological elements are certainly in support of making a more probable diagnosis, for example signs and symptoms of respiratory infection or signs of diffuse inflammation of the parenchyma point more towards an inflammatory and infectious hypothesis. In any case, the most important element remains the short-term time evolution (4–12 weeks), in which an infection generally resolves or worsens rapidly while cancer remains stable or generally increases slightly over a short period of time. In very rare cases, an initial partial resolution of cystic lung cancer may also occur, which hides its cancerous nature.

Cystic airspaces associated with paraseptal emphysema are primarily found in the lung apices, especially in smokers [33]. Moreover, lung apices often show scarring changes, better known as apical capping according to the Fleischner Glossary [24], with the proliferation of extra pleural fat, which can mimic the appearance of peri cystic wall thickening on lung window images. A careful inspection with both lung and soft tissue window settings can help to recognize fat attenuation in the extra pleural space, which explains the apparent thickening of bulla walls in lung window images.

The lungs are also a common site for metastases from other primary cancers [34,35]. These metastases can grow near preexisting cysts, and their progression may resemble the patterns seen in the primary lung cancer associated with cystic airspaces. The presence of a known primary cancer or other metastases in general can help to establish a differential diagnosis. Additionally, treatment for metastatic disease—particularly with antiangiogenic agents—can cause cavitation, potentially leading to changes like the formation of cysts [36].

Evaluating lung nodules or cancers in the context of diffuse pulmonary fibrosis can be particularly challenging. Oh et al. [37] studied 63 patients with early-stage T1 cancers in the setting of interstitial lung fibrosis. They observed a predilection for cancers to develop in the lower lobes, often in close association with fibrotic areas. More than 50% of these cancers were found at the interface between fibrotic cysts and unaffected lung parenchima, while 31% were centrally located within the fibrotic zones. Therefore, thickening or nodularity of cyst walls at the boundary between normal and fibrotic lung tissue should raise suspicion for malignancy [37].

## 4. Other Imaging Assessments

### 4.1. Positron Emission Tomography–Computed Tomography (PET-CT)

Hybrid PET/CT imaging has the advantage of combining functional information with morphological data. The most used PET tracer in oncology is fluorine-18 fluorodeoxyglucose ([^18^F]FDG), a radiolabeled glucose analogue used to evaluate the increased glucose metabolism in cancers. Evidence-based data have demonstrated that [^18^F]FDG PET/CT is able to detect the majority of lung cancers due to their increased glucose metabolism with good diagnostic accuracy values [38]. However, the false positive and false negative findings of [^18^F]FDG PET are well recognized; the first group includes inflammatory/infectious lesions and post-therapeutic changes, while the second group includes non-[^18^F]FDG avid malignancies (such as low-grade adenocarcinoma or typical carcinoids) due to their slow cell proliferation or poor cellularity. Lung cancers presenting as a ground-glass or part-solid nodule, as consolidation or associated to cystic airspaces, may also present low or no significant [^18^F]FDG uptake, resulting in low sensitivity of [^18^F]FDG PET/CT [39]. In particular, the metabolic evaluation of lung cancers associated with cystic airspaces is often limited using [^18^F]FDG PET/CT since the solid component of these cancers are usually small in size, leading to a frequent pitfall; in other words, these cancers are often misinterpreted as benign due to the lack of significant [^18^F]FDG uptake [38,40]. Beyond the qualitative (visual) analysis of [^18^F]FDG PET images, the semi-quantitative analysis using the maximal standardized uptake value (SUV_max_), though widely employed, shows the relevant limitations in discriminating among benign and malignant lung cancers (including those with cystic airspaces) [38,40]. Therefore, a SUV_max_ cut-off to discriminate among benign and malignant lung cancers with cystic airspaces cannot be used in the clinical routine [41]. Conversely, when lung nodules associated with cystic airspaces are incidentally detected by [^18^F]FDG PET/CT, a 6 month follow-up CT chest examination could be recommended to reassess their morphology and growth [39].

### 4.2. Magnetic Resonance Imaging (MRI)

MRI is still considered to be of limited value in lung nodule assessment as compared to CT, especially for long acquisition time (prone to high number of artifacts), low special resolution, and costs. Nodules, masses, and tissues with increased occupancy of the interstitial space (i.e., cancers with increased cellularity) appear hyperintense on T1-weighted and T2-weighted sequences against a suppressed background, increasing its conspicuity, although this presentation is not common and is difficult to interpret as a standalone finding [42]. Recent technological developments, such as the introduction of functional imaging, mainly referring to diffusion-weighted imaging (DWI), have increased the accuracy of diagnostic imaging and prognostication [43] and may warrant some promising results in the future. Indeed, while the spatial resolution of DWI is in the order of millimeters, changes in diffusion can be measured on the micrometer scale and can provide important quantitative functional information [42].

## 5. Tissue Sampling—CT-Guided Biopsy

The need for a precise diagnosis is often needed for the management of thoracic nodules and masses [44]. A pathological specimen may be obtained in clinical practice through endobronchial ultrasound trans-bronchial needle aspiration as well as through CT-guided percutaneous lung biopsies, usually according to the location of the target area within the lung, roughly inner or outer part of the lung, respectively [45,46,47,48]. Recently, a retrospective and single-center study analyzed the diagnostic performance of percutaneous CT-guided biopsies in suspected lung cancers associated with cystic airspaces, both in terms of diagnostic yield and frequency of complications [49]. To date, these authors are the only ones to have considered this issue in detail, and the first conclusions drawn from their analyses are positive, i.e., transthoracic biopsy has an acceptable accuracy in the face of a lower number of complications, which does not exceed that of classic lung cancers. However, the authors themselves acknowledge that their data should be confirmed by larger, possibly prospective, studies, and that the experience of the operator who performs the procedure should also be considered.

## 6. Future Perspectives

A field that needs to be addressed regarding lung cancer associated with cystic airspaces is their relationship with multifocal cancers. Indeed, it is still unclear if the presence of these two entities together increases the likelihood of cancer and if, secondarily, it affects prognosis.

Furthermore, the possibility of aiding detection and interpretation of cystic airspaces through artificial intelligence (AI)-based systems are presently lacking.

Many radiomics studies have been published with the aim of a better prognostication of lung cancer [50,51]; however, the application of different types of AI tools, dedicated filters, or other tools [52] possibly dedicated to cystic airspaces are much less studied. A recent comprehensive review provided an overview of the latest machine and deep learning systems used for detecting airway disorders, including cystic fibrosis, emphysema, lung cancer, mesothelioma, COVID-19, pneumoconiosis, asthma, pulmonary edema, tuberculosis, and pulmonary embolism. The authors found that many models struggled to differentiate between diseases or detect anomalies in the data. Additionally, some models were limited by small datasets, lacked proper data preprocessing, and failed to provide localization information for the final image. These limitations significantly affected the models’ overall performance and need to be addressed. Some approaches, such as random forest and logistic regression, showed poor prediction accuracy due to issues like overfitting and underfitting. Another key issue demonstrated was that many studies focused on just one or two airway diseases, limiting the models’ ability to distinguish between a wider range of airway disorders [53]. Therefore, although many AI-based systems are under investigation for the detection and diagnosis of lung cancer, there is still space for improvement in this field, possibly including the diagnosis and evolution of cystic airspaces.

## 7. Conclusions

Lung cancers associated with cystic airspaces represent a small part of lung cancers, whose data in the literature are relatively scarce. However, as part of the commitment to an early diagnosis of lung cancer, both in the context of dedicated and opportunistic screening, it is important to be aware of it. The importance of these cancers is demonstrated by the latest updates of lung-RADS, which include a dedicated classification of cancers arising in cystic airspaces. The main diagnostic imaging modality to assess the risk of developing lung cancer over a cystic airspace is CT. FDG PET/CT has a minor role, while promising data have recently emerged regarding the performance of the biopsy. However, since most of the data we rely on to interpret these cancers are retrospective, mainly extracted from high risk populations (included in lung cancer screening programs), there are several issues to be explored, including the real incidence and prevalence of cystic airspaces in the overall population, as well as the correct management and follow-up for them, meaning that we should either consider them in a way similar to subsolid nodules or we should create a dedicated management.

## Figures and Tables

**Table 1:** CT findings and management of atypical pulmonary cystic airspaces according to the last Lung RADSv2022 [29].

Lung RADS Category	Findings in Atypical Pulmonary Cysts	Management
3	Growing cystic component (mean diameter) of a thick-walled cys	6-month LDCT
4 A	Thick-walled cyst Multilocular cyst at baseline Thin- or thick-walled cyst that becomes multilocular	3-month LDCT; PET/CT may be considered if there isa ≥ 8 mm (≥268 mm^3^) solid nodule or solid component
4 B	Thick-walled cyst with growing wall thickness/nodularity Growing multilocular cyst (mean diameter) Multilocular cyst with increased loculation or new/increased opacity (nodular, ground glass, or consolidation	Diagnostic chest CT with or without contrast; PET/CT may be considered if there isa ≥ 8 mm (≥268 mm^3^) solid nodule or solid component; tissue sampling; and/or referral for further clinical evaluation Management depends on clinical evaluation, patient preference, and the probability of malignancy

## Data Availability

Not Applicable.
